# The Genus *Lagochilus* (Lamiaceae): A Review of Its Diversity, Ethnobotany, Phytochemistry, and Pharmacology

**DOI:** 10.3390/plants10010132

**Published:** 2021-01-11

**Authors:** Nilufar Z. Mamadalieva, Davlat Kh. Akramov, Ludger A. Wessjohann, Hidayat Hussain, Chunlin Long, Komiljon Sh. Tojibaev, Elham Alshammari, Mohamed L. Ashour, Michael Wink

**Affiliations:** 1 Institute of the Chemistry of Plant Substances of the Academy Sciences of Uzbekistan, Mirzo Ulugbek Str 77, Tashkent 100170, Uzbekistan; a.davlat@inbox.ru; 2 Department of Bioorganic Chemistry, Leibniz Institute of Plant Biochemistry, Weinberg 3, 06120 Halle (Saale), Germany; ludger.wessjohann@ipb-halle.de (L.A.W.); hidayat.hussain@ipb-halle.de (H.H.); 3 College of Life and Environmental Sciences, Minzu University of China, Beijing 100081, China; long@mail.kib.ac.cn; 4 Key Laboratory of Ethnomedicine, Ministry of Education, Minzu University of China, Beijing 100081, China; 5 Institute of Botany of the Academy Sciences of Uzbekistan, Durmon Yuli Str 32, Tashkent 100125, Uzbekistan; ktojibaev@mail.ru; 6 Department of Pharmacy Practice, College of Pharmacy, Princess Nourah bint Abdulrahman University, Riyadh 11671, Saudi Arabia; ejalshammari@pnu.edu.sa; 7 Department of Pharmaceutical Sciences, Pharmacy Program, Batterjee Medical College, Jeddah 21442, Saudi Arabia; ashour@pharma.asu.edu.eg; 8 Department of Pharmacognosy, Faculty of Pharmacy, Ain Shams University, Cairo 11566, Egypt; 9 Department of Pharmaceutical Biology, Institute of Pharmacy and Molecular Biotechnology, Heidelberg University, 69120 Heidelberg, Germany

**Keywords:** *Lagochilus*, Lamiaceae, diversity, traditional medicine, phytochemistry, secondary metabolites, pharmacology, biological properties

## Abstract

The genus *Lagochilus* (Lamiaceae) is native to Central, South-Central, and Eastern Asia. It comprises 44 species, which have been commonly used as herbal medicines for the treatments of various ailments for thousands of years, especially in Asian countries. This review aims to summarize the chemical constituents and pharmacological activities of species from the genus *Lagochilus* to unveil opportunities for future research. In addition, we provide some information about their traditional uses, botany, and diversity. More than 150 secondary metabolites have been reported from *Lagochilus*, including diterpenes, flavonoids, phenolic compounds, triterpenoids, iridoid glycosides, lignans, steroids, alkaloids, polysaccharides, volatile, non-volatile and aromatic compounds, lipids, carbohydrates, minerals, vitamins, and other secondary metabolites. *In vitro* and *in vivo* pharmacological studies on the crude extracts, fractions, and isolated compounds from *Lagochilus* species showed hemostatic, antibacterial, anti-inflammatory, anti-allergic, cytotoxic, enzyme inhibitory, antispasmodic, hypotensive, sedative, psychoactive, and other activities.

## 1. Introduction

Terrestrial plants have been used for centuries as an endless well for supplying many medicinally important secondary metabolites that are used effectively in curing various ailments. They are considered as the milestone of all the traditional medicinal systems used throughout the whole world since antiquity. This fact attracted researchers in drug discovery to explore these plants frequently with the aim of finding new secondary metabolites or to validate the claimed ethnopharmacological uses.

Family Lamiaceae (the mint family) is among the largest family that contains about 236 genera and 6900 to 7200 species. The family is famous for many of its widely used genera including *Salvia*, *Scutellaria*, *Stachys*, *Plectranthus*, *Hyptis*, *Teucrium*, *Vitex*, *Thymus,* and *Nepeta*. Members from the Lamiaceae are important ornamental, medicinal, and aromatic plants and have been used as traditional herbal medicines for thousands of years [1]. They are also used as culinary herbs, spices, and vegetables and as ingredients in cosmetics, hygienic products, and perfumes [2]. 

The genus *Lagochilus* is a small genus that comprises about 44 species that are native to Central, South-Central, and Eastern Asia [3,4]. It is highly drought-tolerant and is considered a typical mountain plant. Most of these species have been commonly used as herbal medicines for the treatment of various ailments for thousands of years, especially in Asian countries. Species of this genus exhibit various pharmacological effects, such as hemostatic, antispasmodic, and anti-edemic properties, and can be used against bleeding, coronary heart disease, chest pain, skin conditions, stomach pain, and as a tranquilizer. The most reported properties include relaxation, insomnia, dementia, euphoria, and subtle perceptual changes. *Lagochilus* is also used for the treatment of allergies and skin diseases [5,6,7,8,9]. Pharmacological studies indicated that mainly diterpenoids have hemostatic abilities [10,11].

Previous chemical instigations on the genus are limited due to the fact that many species are classified endangered, and the collection of these plants is not easy. However, this effort on the chemical profiling of the plants belonging to the genus led to the isolation and characterization of many diterpenes, flavonoids, iridoids, triterpenes, and polysaccharides. The diterpene lagochiline and its derivatives are among the promising hemostatic agents [5,7,10,11].

To the best of our knowledge, no reviews in the literature provide comprehensive insights into the medicinal uses and importance of *Lagochilus* plants. Although there is some work done about limited species that showed the very promising potential of the studied plants nevertheless, many other species of the genus remain untouched. Therefore, this review presented an effort to summarize all the up-to-date published data about the genus and provided comprehensive and condensed references dealing with all the required data about botanic characterization, distribution, traditional uses, primary and secondary metabolites, and pharmacological activities of the undiscovered genus *Lagochilus*. This review might shed light on the unseen genus that might attract researchers in drug discovery to find new compounds with promising biological potential.

## 2. Materials and Methods

The literature for this review was collected by searching various scientific electronic databases, including GoogleScholar (https://scholar.google.com/), PubMed (https://pubmed.ncbi.nlm.nih.gov/), SpringerLink (https://link.springer.com), SciFinder (https://scifinder.cas.org/), ScienceDirect (https://www.sciencedirect.com/), and Web of Science (https://mjl.clarivate.com), via searching of these keywords: “*Lagochilus* and phytochemistry”, “*Lagochilus* and chemical compounds”, “*Lagochilus* and traditional uses”, and “*Lagochilus* and biological activity” in literature written in English, Russian, and Uzbek. Additional knowledge from other sources of literature was extracted (books, thesis). Altogether, about 94 references covering the isolation of phytoconstituents, as well as the diversity, traditional uses, and biological properties of the *Lagochilus* genus species from 1954 to 2020, were chosen unbiased to be reviewed in the work. The Plant List (http://www.theplantlist.org/), International Plant Name Index (https://ipni.org/), and Kew Botanical Garden Plant name (https://wcsp.science.kew.org/home.do) databases were used to validate the scientific name.

The review is built on giving the general botanical characteristics of the genus, followed by distribution of the species worldwide and a compilation of the traditional uses. Then the chemical isolated secondary metabolites are reported in subclasses based on their chemical structures, and finally, the biological work was subcategorized based on uses. 

## 3. Taxonomy and Botany

**Figure 1:**
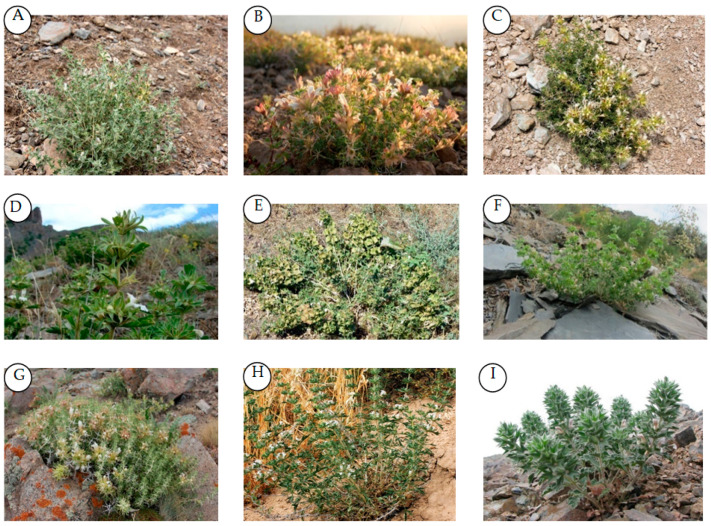
Photograph for some *Lagochilus* species from Uzbekistan Flora (**A**): *L. inebrians* Bunge, (B): *L. nevskii* Knorring, (**C**): *L. occultiflorus* Rupr., (**D**): *L. olgae* Kamelin, (**E**): *L. platycalyx* Schrenk, (**F**): *L. proskorjakovii* Ikramov, (**G**): *L. seravschanicus* Knorring, (**H**): *L. setulosus* Vved., (**I**): *L. vvedenskyi* R. Kam. (Photos A, B, D, G are taken by Natalya Beshko, photos C, H are taken by Alim Gaziev, photo E is taken by Tulkin Tillaev, photos F, I are taken by Akbar Akhmedov).

Within the Lamiaceae family, *Lagochilus* belongs to the subfamily Lamioideae and tribe Leonureae Dumort [12]. Species of *Lagochilus* are subshrubs or perennial herbs (Figure 1). Rootstocks are woody. Stems are green-white, rigid, and sparsely hirsute. The leaf blade is rhombic, palmatipartite, or pinnatipartite, with lobes spinescent, sometimes subtending sterile spinescent bracteoles. Normally, 2–10 flowers grow vertically. The calyx is campanulate to tubular-campanulate, 5-veined; throat oblique, straight; teeth five, subequal or three posterior teeth longer, triangular to oblong or broadly ovate, usually longer than the tube, apex spinescent. Corolla is villous outside, pilose annulate inside, 2-lipped; upper lip oblong, straight, slightly concave, 2-lobed or 4-toothed; lower lip obliquely spreading, 3-lobed; middle lobe largest, obcordate, 2-lobulate; lateral lobes straight, acute, or emarginate. Stamens are four, exserted or sub included, anterior two longer; with filaments complanate; anther cells two, parallel or divergent, ciliate. Style is filiform, apex subequally 2-cleft. Nutlets are flattened-obconical, oblong–obovoid or oblong–ovoid, apex truncate or rounded, glandular, dusty hairy, scaly, or glabrous, smooth.

## 4. Diversity

The genus *Lagochilus* is mostly distributed on dry slopes, in valleys, and deserts from Iran to Mongolia, Russia (south Siberia), northwest China, north Pakistan [1] and has a distributional center in Tianshan Mountains and Central Asia. The results of the studies of Zhang et al. [3] showed that the Tianshan Mountains, especially the western Ili-Kirghizia Tianshan, as well as Sunggar and Kaschgar, were the ancestral area. The ancestral biome was mainly in the montane steppe zone of valley and slope at altitudes of 1700 ± 2700 m above sea level, and the montane desert zone of foothill and front-hill at 800 ± 1700 m. The main center of diversity lies in Central Asia. According to Tskervanik [12], in world flora, there are 44 species that can be distinguished in *Lagochilus*. According to the taxonomy of this author, 34 species grow in the territory of the CIS (Commonwealth of Independent States). 

In the flora of Uzbekistan, this genus is represented by 18 species (Table 1) [13]. Taxa of the genus *Lagochilus* basically occur throughout the territory of Uzbekistan, starting from the deserts to Tian-Shan and Pamir-Alay mountain systems. The majority of species can be found in the Pamir-Alai Mountain, south-west of Tian-Shan and Turanian lowland [13]. Species from the genus *Lagochilus* belong to the most vulnerable plant species from the Lamiaceae family. Out of the existing 18 *Lagochilus* species in the flora of Uzbekistan, four are included in the Red Book of the Republic of Uzbekistan [14]: *L. vvedenskyi*, *L. olgae*, *L. proskorjakovii*, and *L. inebrians*. The main population of these species grows in Nuratau and Kyzylkum deserts. On the basis of occurrence, these Red List plant species belong to category I (disappearing) and II (rare species). In addition, the natural distribution of other species from this genus, such as *L. gypsaceus* and *L. acutilobus,* are also limited across the country [15]. In the territory of South Kazakhstan, 10 species of *Lagochilus* were found, and the areas of these species are located in Karatau, Karzhantau, and Aksu-Zhabagly [16]. In the Flora of China, there are 14 *Lagochilus* species distributed wildly in the northwest region. One of the species, *L. ilicifolius*, grows in the desert (sandy land) and steppe in northwestern China (Neimeng, Gansu, Shanxi, and Ningxia provinces), whereas the other 13 species are distributed mainly in the Xinjiang region [3,10,17]. The Flora of Iran comprises six species, and four of them (*L. macranthus* Fisch and C.A. Mey., *L. quadridentatus* Jamzad, *L. lasiocalyx* (Stapf) Jamzad, and *L. aucheri* Boiss.) are endemic to Iran [4,18,19]. Three species of *Lagochilus* are found in Mongolian flora, and they are widely distributed in the Khangai, Mongolian Altai, Middle Khalkha, Depression of Great Lakes, Valley of Lakes, East Gobi, Gobi-Altai, Transaltai Gobi, Alashan Gobi regions (Table 1) [20,21].

## 5. Traditional Uses

The traditional use of *Lagochilus* plants dates back centuries. *L. inebrians* is commonly known as Inebriating Mint, Intoxicating Mint, Turkistan Mint, or Intoxicating Hare’s Lip. The name *L. inebrians* is derived from the Greek words “lagos” and “cheilos”, literally meaning “hare” and “lip/cheek” and “inebrians”, meaning intoxicating, thus translating to “intoxicating hare’s lip” (Wikipedia). A decoction of herb and roots of *Lagochilus* species are used in folk medicine as a styptic and also against skin conditions, stomach pain, and as tranquilizers. Many species of the *Lagochilus* genus have been used in traditional medicine to treat hemorrhages and inflammation [8,9]. The most commonly reported effects include relaxation, euphoria, and subtle perceptual changes. It is also used for the treatment of allergies and skin diseases. In Central Asia, *L. inebrians* has been used during celebrations for its intoxicating and sedative effects [22]. People make infusions with the dried leaves of *L. inebrians;* honey and sugar are often added to this drink to reduce its bitter taste. A summary of their traditional use is presented in Table 2.

## 6. Phytochemical Studies 

Phytochemical studies performed on different *Lagochilus* species have demonstrated the occurrence of several classes of primary and secondary metabolites. A literature survey revealed that the genus *Lagochilus* is a rich source of diterpenoids [30,31,32,33], flavonoids [29,34], phenolic compounds, triterpenoids [34], iridoid glycosides, lignans, steroids, alkaloids, polysaccharides [35], volatile, non-volatile and aromatic compounds, lipids, carbohydrates, minerals, vitamins, and others. About 150 secondary metabolites have been reported from all previous classes in the genus. Among these compounds, diterpenoids have been considered as useful taxonomic markers and the major active components of *Lagochilus* species. In the following section (Table 3), we will summarize the reported natural products isolated from each species. Out of 44 species presented in the genus, only 19 species have been phytochemically explored. Therefore, the genus still holds more than half of its species unseen by researchers.

In the following part, we will classify the isolated compounds from the species based on their chemical structures. The most relevant citation about the isolation and characterization of these secondary metabolites will be given and highlighted.

### 6.1. Diterpenes

Diterpenes are the most characteristic and important phytochemicals in *Lagochilus*, the highest numbers of which were reported in *L. inebrians* [48], *L. pubescens* [60], and *L. platyacanthus* [26] (Table 4, Figure S1). Lagochilin (**1**) is the main component of the total extractive substances of many species of the genus. Lagochilin (**1**) identified in *L. inebrians, L. setulosus, L. gypsaceus* [37], *L. hirsutissimus* [33], *L. proskorjacovii* [61], and *L. pubescens* [62]. *L. inebrians* gathered in Samarkand province contained 2.3% lagochilin, *L. setulosus* gathered in Chimkent province 1.1%, and *L. gypsaceus* gathered in Kashkadar’ya province 2.1% [66]. Lagochirsine (**16**) is found free in the three plant species *L. hirsutissimus, L. setulosus,* and *L. gypsaceus*. However, its content in these plants was 0.2–0.3% [39].

### 6.2. Flavonoids and Phenolic Glycosides

*Lagochilus* species are characterized by rich biological activities, such as antioxidant, anti-allergic, and enzyme inhibitory activities due in part to the presence of a diversity of flavonoids and phenolic compounds [21,27,28,29,40,50,58]. Flavonoids accumulate in different plant parts of the *Lagochilus* species. It is believed that most flavonoids in the *Lagochilus* are derivatives of quercetin, luteolin, and kaempferol. To date, about 60 flavonoids have been identified in *Lagochilus*. The structural formulas of major flavonoids and their glycosides isolated or identified from raw materials of *Lagochilus* species are shown in Table 5 and Figure S2.

### 6.3. Iridoids and Their Glycosides

Iridoids are a type of monoterpenoids in the general form of cyclopentanopyran and are found in a wide variety of plants as glucosides. 8-O-Acetylharpagide (**99**) and harpagide (**100**) are common iridoids in *Lagochilus* [6,50,52]. The latest results of our investigations showed that *L. inebrians* and *L. gypcaseus* are rich for 8-O-acetylharpagide [40,50]. However, only a few (seven) iridoids could be reported in the genus. From the aerial parts of *L. ilicifolius* ajugoside (**101**), ajugol (**102**), geniposidic acid (**103**), mussaenosidic acid (**104**), and 8-deoxyshanzhiside (**105**) were identified and isolated [5,58] (Table 3, Figure S3).

### 6.4. Essential Oils 

The species of *Lagochilus* are aromatic with a pleasant scent. Germacrene D (**107**), α-pinene (**108**), and β-bourbonene (**109**) are the main components of the oil of *L. aucheri* [18]. In the oil of *L. cabulicus* α-pinene (**108)**, *β*-springene (**110**), and geranyllinalool (**111**) are major compounds [23]. The oil of *L. macranthus* mainly contains the oxygenated sesquiterpenes caryophyllene oxide (**112**), humulene epoxide II (**113**), and viridiflorol (**114**) [56]. The major constituents of *L. kotschyanus* appear to be α-pinene (**108)**, myrcene (**115**), and *β*-caryophyllene (**116**), respectively [19]. In the oil of *L. diacanthophyllus,* the main components are α-pinene (**108**) and dillapiol (**117**) [67,68]. The chemical composition of three *Lagochilus* species essential oils: *L. gypsaceus*, *L. inebrians*, and *L. setulosus*, were determined using the GC-MS method [69]. The results showed that the studied essential oils are made up mainly of linalool, β-ionone, trans-chrysanthenyl acetate, α-terpineol for *L. gypsaceus*; trans-chrysanthenyl acetate, eugenol, trans-verbenol, bicyclo [3.1.1]hept-3-en-2-one, pinocarvone for *L. inebrians*; and finally 2,4-bis(1,1-dimethylethyl)phenol, bicyclo[3.1.1]hept-2-en-4-ol, hexadecanoic acid, limonene, 2-hexenal for *L. setulosus*. Our results indicated that limonene, furfural, benzaldehyde, 4-terpineol, myrtenal, α-terpineol, myrtenol, and *p*-cymen-7-ol are the common compounds to these species. Monoterpens linalool, trans-chrysanthenyl acetate, α-terpineol, eugenol have been reported as the most abundant compounds in the oil of *L. gypsaceus* and *L. inebrians,* while *L. setulosus* oil consists of aliphatic alcohol, aldehyde, and ketons. Especially, oxygenated monoterpenes trans-chrysanthenyl acetate and eugenol were found for the first time in substantial amounts in the oil from *L. gypsaceus* and *L. inebrians*. A comparison of our studies confirms that the dissimilarities in the composition of the essential oils of three *Lagochilus* species may be considered as an indication of several chemotypes existing within the genus (Table 3, Figure S4).

### 6.5. Triterpenes

Plant triterpenoids represent a large and structurally diverse class of natural products. However, they represent a minor class of compounds reported from *Lagochilus* species. Only erythrodiol (**118**) from *L. lanatonodus* [27], oleanolic (**119**), and ursolic acid (**120**) from *L. leiacanthus* [28] were isolated (Table 3, Figure S5).

### 6.6. Steroids

Plant steroids are a diverse group of secondary metabolites. Up to now, phytochemical studies have afforded six steroids from the *Lagochilus* genus, which sitosteryl acetate (**122**), stigmasteryl acetate (**124**), lupeol (**126**) from *L. cabulicus* [23,36] (Table 3, Figure S6). *L. gypsaceus* afforded daucosterol (**125**) and β-sitosterol (**121**) [40]. β-Sitosterol (**121**), stigmasterol (**123**), and daucosterol (**125**) were isolated from *L. inebrians* [50]. β-Sitosterol (**121**) was identified in *L. lanatonodus* and *L. pubescens* [12,27,30,51,60,61].

### 6.7. Alkaloids

Alkaloids are a group of important secondary metabolites. Alkaloids and extracts of alkaloid-containing plants have been used throughout human history as remedies, poisons, and psychoactive drugs. Stachydrine has been identified in many Lamiaceae species [53,54]. Multiple studies have confirmed that stachydrine has strong neuroprotective, anti-fibrotic, and anti-inflammatory effects [70]. Stachydrine (**127**) was reported in *L. inebrians* and *L. hirsutissimus* [42,54,63]. 3-Methyl-1,2,3,4-tetrahydroquinoline (**128**), 4-hydroxyisoquinoline (**129**), songoramine (**130**), and songorine (**131**) were determined from *L. ilicifolius* [21] (Table 3, Figure S7).

### 6.8. Lignans

The lignans are fiber-associated compounds found in plants, particularly seeds, nuts, grains, and vegetables. Lignans are rare in the *Lagochilus* genus, with only three lignans named erythro-1-[(4-O-β-D-glucopyranosyl-3-methoxyl)- phenyl]-2-[(5’-methoxyl)-pinoresinol]-propane-1,3-diol (**132**), tortoside C (**133**), and sisymbrifolin (**134**) isolated from *L. ilicifolius* [7] (Table 3, Figure S8). Another three lignans: 1-(4-hydroxy-3-methoxy)-phenyl-2-[4-(1, 2, 3-trihydroxypropyl)-2-methoxy]-phenoxy- 1, 3-propandiol (**135**), (+)-isolarisiresinol 3-α-O-β-D-glucopyranoside (**136**), and (-)-isolarisiresinol 3-α-O-β-D-glucopyranoside (**137**) were isolated from whole herb of *L. platyacanthus* [58]. From *L. ilicifolius* (+)-syringaresinol (**138**) was identified by Qian et al. [7].

### 6.9. Aliphatic Alkanes and Alcohols

Plants have an outer surface layer of wax, which is usually a complex mixture of aliphatic lipid compounds, such as straight and branched chain alkanes, alkenes, long-chain fatty acids and esters, long-chain fatty alcohols, long-chain fatty aldehydes, and ketones. Nonacosane (**139**) (in *L. inebrians, L. proskorjacovii, L. pubescens*) [12,51,60,61], hentriacontane (**140**), tritriacontane (**141**) (in *L. inebrians*) [51], phytol (**142**), 12-hentriacontanol (**143**), and octacosanol (**144**) (in *L. ilicifolius*) [5], dacosyl ester (**145**) (in *L. lanatonodus*) [27] are present in *Lagochilus* species (Table 3, Figure S9).

### 6.10. Lipids

In plants, lipids play especially important roles as signaling and energy storage compounds [55]. Lipid compounds were identified and analyzed in *Lagochilus* species by some researchers [41,55,57]. Laballenic (**146**), octadeca -5,8-dienoic (**147**), eicos -11-enoic (**148**), and eicosa-9,11-dienoic acids (**149**) were detected in *L. occultifloris* [57] (Table 3, Figure S10). Free and polar lipids from ripe seeds of *L. inebrians* were investigated by Yuldasheva et al. [55], and the findings showed that seeds have a very high percentage of oil content, a large fraction of unsaturated ω-3 and ω-6 fatty acids.

### 6.11. Miscellaneous Compounds

Some other metabolites were also discovered in this genus, such as scopoletin (**150**) [7,27,41], ascorbic acid (**151**), β-caroten (**152**) [63] (Figure S11). The following class of metabolites were identified in *Lagochilus* species: carbohydrates [35,64], sugars [63], polysaccharides, pectin, hemicellulose [64,65,71], tannins [63], organic acids, resins [63], and microelements [63,72,73].

## 7. Biological Activities

The biological activities, including hemostatic, antibacterial, anti-inflammatory, anti-allergic, cytotoxic, enzyme inhibitory, antispasmodic, sedative, hypotensive, psychoactive, and other activities, have been reported in some publications. Different biological activities have been reported from the extracts, fractions, essential oils, and their isolated compounds. Mostly, *Lagochilus* species have been used as a folk medicine for treating hemostatic [10,11,25,35,39,58,64,65,71,74,75,76,77,78,79,80,81,82,83,84,85], inflammation [10,85,86,87], and ulcers [7] in Chinese and Central Asian traditional medicine. Reported biological activities of the genus *Lagochilus* are written in subcategories below.

### 7.1. Hemostatic Effect

The plants of the genus *Lagochilus* have been known for their therapeutic effects and have been included in medicinal practice as valuable hemostatic agents [10,11,25,35,39,58,64,65,71,74,75,76,77,78,79,80,81,82,83,84,85]. The mechanism of the hemostatic action of *Lagochilus* preparations was evaluated using different *in vivo* tests (Table 6). The extracts of *Lagochilus* species have shown a promising activity when applied to treat hemophilia. In China and Central Asian countries, the species of *Lagochilus* are widely used as effective hemostatic agents.

### 7.2. Anti-Inflammatory Activity

Some species of the *Lagochilus* genus have been used in Chinese traditional medicine to treat inflammation. The results of anti-inflammatory activities of the components from *Lagochilus* species are summarized in Table 7 [10,85,86,87]. This could be due to iridoid glucosides, which have been shown to be potent anti-inflammatory compounds [10,85].

### 7.3. Antibacterial Activity

The antibacterial effect of the components (different extracts, essential oils, total alkaloids, and some individual compounds) from *Lagochilus* species were evaluated [5,6,7,19,21,24,88]. The results indicated that the components of *L. ilicifolius, L. cabulicus, L. acutilobus, L. gypsaceus, L. inebrians, L. olgae, L. proskorjakovii, L. setulosus, L. vvedenskyi* had weak antibacterial effects on human pathogens (Table 8). However, Taban et al. [19] reported that the essential oil obtained from the flowers of *L. kotschyanus* showed strong inhibitory activity against Gram-positive bacteria *S. pyogenes, S. agalactia,* and *B. anthracis* (MIC 3.1 mg/mL).

### 7.4. Antioxidant Activity

The antioxidant activity of methanol extracts of seven *Lagochihis* species was evaluated using a metal chelating (MC), phosphomolybdenum (PPBD), ferric reducing power (FRAP), cupric reducing antioxidant capacity (CUPRAC), 2,2’-azino-bis (3-ethylbenzothiazoline-6-sulphonic acid) (ABTS), and 2,2-diphenyl-1-picrylhydrazyl (DPPH) assays. In these assays, we reported the antioxidant activities as Trolox equivalents, whereas ethylenediaminetetraacetic acid (EDTA) was used for metal chelating assay [40]. Regarding quenching of DPPH radical activity, the observed abilities decreased in the order: *L. inebrans* (collected from Djizzakh)>*L. vvedenskyi*>*L. olgae*>*L. setulosus*>*L. proskorjakovii*>*L. gypsaceus*>*L. acutilobus*>*L. inebrans* (collected from the Surkhandarya region). Similar to DPPH, the best cupric (CUPRAC) and ferric reducing power (FRAP) ability were determined by *L. inebrans* (from Djjzzakh), followed by *L. vvedenskyi*. The antioxidant activity is probably due to the polyphenols present. The highest amount of total phenolic compounds was found in *L. inebrans*, followed by *L. vvedenskyi* and *L. proskorjakovii,* and we observed a strong correlation between total phenolic content and antioxidant (DPPH, CUPRAC, and FRAP) properties of the tested extracts. In the PPBD assay, *L. proskorjakovii* exhibited the strongest ability with 2.00 mmolTE/g, while *L. inebrians* (from Surkhandarya) was the weakest. In the ferrozine assay, the MC ability of *L. acutilobus* was the best, followed by *L. olgae* and *L. setulosus*. We also evaluated the antioxidant potential of 3 *Lagochilus* essential oils mentioned above in vitro assays [69]. *L. inebrians* oil exhibited the best antioxidant effect in these assays. For example, *L. inebrians* had about a 16-fold higher activity than that of *L. setulosus* in DPPH assay. Similarly, *L. inebrians* exhibited a significant reducing power effect with 2093.33 mgTE/g in FRAP assay. The second highest antioxidant value was noted in *L. setulosus,* except for the MC assay. The observed activity for *L. inebrians* may be explained with its chemical profiles. The main components (*trans*-chrysanthenyl acetate, eugenol, and verbenol) have already been reported as strong antioxidant compounds, and they could be considered as the main contributors to the observed activity [69]. On the other hand, only very rare work could be traced regarding the *in vivo* antioxidant activity of the plants belonging to the genus *Lagochilus*. Only three species were examined by Jiao et al. [10] for their effect on inducible nitric oxide synthase, malondialdehyde, and superoxide dismutase. In this study, both aqueous and alcohol extracts of *L. lanatonodus* were able to inhibit the enzymes at 200 and 400 mg/kg doses similar to aspirin (positive control).

### 7.5. Anti-Allergic Activity

*Lagochilus leiacanthus* is a folk medicine used for the treatment of inflammation and ulcers in Xinjiang, China. Mast cells and basophils play a central role in allergic and inflammatory reactions. The β-hexosaminidase release from RBL-2H3 (rat basophilic leukemia) mast cells was measured as an indicator of the inhibitory activity against allergic reactions. In biological studies on the alcoholic extracts of the whole herb of this plant, Furukawa et al. [28] found that the extract inhibits the release of β-hexosaminidase from RBL-2H3 cells. They also examined the inhibitory activity of the isolated compounds. Among the isolated compounds, flavanones **51–52** and flavones **54**, **56**, **61**, **65–67** showed inhibition of β-hexosaminidase release more potently than ketotifen used as a positive control. It is worth mentioning that no work could be found regarding the *in vivo* anti-allergic effect of *Lagochilus* species, although most of these plants showed promising anti-inflammatory activity. This point might present a future point for many researchers.

### 7.6. Cytotoxic Activity

The whole herb of *L. ilicifolius* has been used as a folk medicine for treating hemostatic, inflammation, and ulcers in China. The cytotoxic activities of three lignans **132–134** isolated from *L. ilicifolius* were evaluated using the MTT assay against the PC12 cell line derived from rat adrenal pheochromocytoma [7]. *Erythro*-1-[(4-O-β-D-glucopyranosyl-3-methoxyl)-phenyl]-2-[(5’-methoxyl)-pinoresinol]-propane -1,3-diol (**132**) exhibited strong cytotoxic activity against PC12 cell line with an IC50 value of 1.22 ± 0.03 μmol/L, while sisymbrifolin and tortoside C was not cytotoxic.

### 7.7. Enzyme Inhibition

Urease plays an important role in the pathogenesis of many illnesses, such as pyelonephritis, hepatic coma, and peptic ulceration. Tyrosinase plays an important role in melanogenesis (i.e., biosynthesis of melanin pigments, also known as pigmentation). Amylase is an enzyme produced primarily by the pancreas and the salivary glands to help digest carbohydrates. Acetylcholinesterase (AChE) is one of the most efficient enzymes of the nervous system. AChE inhibitors are employed in the treatment of Alzheimer’s disease, myasthenia gravis, glaucoma, smooth muscle atony, and assorted disorders of autonomic nervous system functions. The results of inhibitory enzyme activities of the components from *Lagochilus* species are summarized in Table 9 [34,40,69].

### 7.8. Acute Toxicity

Oral administration of *Lagochilus* extracts, including aqueous and ethanol extracts from *L. lanatonodus, L. diacanthophyllus, L. platyacanthus, L. hirtus,* and *L. ilicifolius*, did not show visible signs of toxicity in mice at the highest dose of 5000 mg/kg of body weight [10]. An acute toxicity study was performed in mice according to the Organization for Economic Cooperation and Development (OECD/OCED) guidelines 423 (Acute Oral Toxicity-Acute Toxicity Class Method) [89] with each group comprising of ten mice. The animals were orally administered a dose of 5000 mg/kg aqueous and ethanol extracts from five *Lagochilus* species and were observed continuously for 30 min and 4 h, intermittently for 24 h, and then once a day for the next 14 days for general behavioral change, signs of toxicity and mortality. There was no evidence of differences in physiological or behavioral responses between the normal control group and extract-treated groups and also no differences in the consumption of food and water, which implied that the five *Lagochilus* species are not toxic.

### 7.9. Other Activities

Preliminary biological investigations on *Lagochilus* components (extracts, fractions, and individual compounds) have indicated their antispasmodic [90], sedative and hypotensive [29], psychoactive [91], and other activities. *Lagochilus* components may be valuable in the treatment of glaucoma [92], dermoplasty [93], hypertension, heart failure, and angina [29,94].

*Lagochilus* species have been traditionally and ethnopharmacologically used to treat many diseases, such as hemorrhages, inflammation, allergies, skin diseases, and stomach pain. The phytochemical and biological results validate and support the ethnopharmacological use of *Lagochilus* species in traditional medicine. Various chemical constituents have been isolated and identified in different groups of compounds, e.g., flavonoids, diterpenes, terpenoids, steroids, iridoids, polysaccharides, and other compounds. Diterpenes and flavonoids are the main bioactive compounds in *Lagochilus* species, and a wide spectrum of biological studies have focused on these compounds. However, the biological effects of other compounds, such as terpenoids, steroids, iridoids, polysaccharides isolated from this genus, have mostly not been investigated. In certain cases, only plant extracts have been tested for biological activities. *Lagochilus* species are a valuable medicinal resource with specific pharmacological activities *in vitro* and *in vivo* studies; however, more comprehensive studies on their pharmacokinetics, metabolism, side effects, and toxicity are required to demonstrate the efficacy and safety of plant extracts or bioactive compounds from this genus.

## 8. Conclusions

We conclude that the genus *Lagochilus* is a good source of phytochemical diversity. This genus is native to Central, South-Central, and Eastern Asia and is mainly distributed in arid and semiarid regions of China, Kazakhstan, Uzbekistan, Mongolia, Iran, Pakistan, Afghanistan, and endemic to the Asian mountains. The obtained results demonstrated that not even the well-studied species of *Lagochilus* had been exhaustively investigated for secondary metabolites, and it should certainly be worthwhile to explore them for new bioactive molecules further. Some species of *Lagochilus* are rare in nature and endangered. Due to the rapid economic development of regions, climate change, over-collection by people, and continuing resource exploitation, some species in this genus face severe threats, and serious efforts are needed to save and protect rare and endangered species from overharvesting and extinction. Efforts should, therefore, be made to cultivate and naturalize the economically relevant species.

## Figures and Tables

**Table 1:** Diversity and distribution of different *Lagochilus* species in Asia.

Distribution	Region	Species	Reference
Afghanistan	western Himalaya	*L. cabulicus* Benth., *L. cuneatus* Benth., *L. hindukushi* Kamelin and Gubanov*, L. schugnanicus* Knorring	https://wcsp.science.kew.org/
China	northwestern China, Xinjiang region (Ili Valley, the Karakoram and Altai Mountains)	*L. ilicifolius* Bunge ex Benth., *L. grandiflorus* C. Y. Wu and Hsuan, *L. platyacanthus* Rupr., *L. kaschgaricus* Ruprecht, *L. diacanthophyllus* (Pall.) Benth, *L. hirtus* Fisch. and C.A. Mey., *L. bungei* Benth*., L. macrodontus* Knorring, *L. kaschgaricus* Rupr., *L. lanatonodus* C.Y. Wu and S.J. Hsuan, *L. leiacanthus* Fisch. and C.A. Mey., *L. pungens* Schrenk, *L. xianjiangensis* G.J. Liu	[3,10,17] https://wcsp.science.kew.org/
Iran		*L. alutaceus* Bunge., *L. cabulicus* Benth., *L. macranthus* Fisch and C.A. Mey., *L. quadridentatus* Jamzad, *L. lasiocalyx* (Stapf) Jamzad, *L. aucheri* Boiss.	[4,18,19]
Kazakhstan	Karatau, Karzhantau, Aksu-Zhabagly	*L. acutilobus* (Ledeb.) Fisch. and C.A. Mey., *L. bungei* Benth.*, L. longidentatus* Knorr., *L. pulcher* Knorr., *L. taucumensis* Zucker., *L. inebrians* Bunge, *L. androsswii* Knorr., *L. leiacanthus* Fisch. et Mey, *L. pungens* Schrenk, *L. hirtus* Fisch. et Mey, *L. diacanthophillus* Benth., *L. kaschgaricus* Rupr., *L. knorringianus* Pavlov, *L. occultiflorus* Rupr., *L. platyacanthus* Rupr., *L. platycalyx* Schrenk ex Fisch. and C.A. Mey, *L. seravschanicus* Knorring, *L. setulosus* Vved., *L. subhispidus* Knorring	[16] https://wcsp.science.kew.org/
Kyrgyzstan	Tian-Shan and Pamir Alai Mountains	*L. diacanthophyllus* (Pall.) Benth*, L. drobovii* Kamelin and Tzukerv*., L. hirsutissimus* Vved*, L. kaschgaricus* Rupr., *L. knorringianus* Pavlov, *L. occultiflorus* Rupr., *L. paulsenii* Briq., *L. platyacanthus* Rupr., *L. platycalyx* Schrenk ex Fisch. and C.A. Mey, *L. pubescens* Vved., *L. pulcher* Knorring, *L. schugnanicus* Knorring, *L. turkestanicus* Knorring	https://wcsp.science.kew.org/
Mongolia	Mongolian Altai and Khangai Mountains, Gobi regions	*L. bungei* Benth., *L. diacanthophyllus*, *L. ilicifolius* Bge	[20,21]
Pakistan	Western Pakistan	*L. cabulicus* Benth.*, L. cuneatus* Benth., *L. schugnanicus* Knorring	https://wcsp.science.kew.org/
Tajikistan	Pamir-Alay Mountains	*L. botschantzevii* Kamelin and Tzukerv., *L. gypsaceus* Vved., *L. hirsutissimus* Vved, *L. inebrians* Bunge, *L. knorringianus* Pavlov*, L. kschtutensis* Knorring, *L. nevskii* Knorring, *L. paulsenii* Briq., *L. platyacanthus* Rupr, *L. platycalyx* Schrenk ex Fisch. and C.A. Mey*, L. pubescens* Vved, *L. schugnanicus* Knorring, *L. seravschanicus* Knorring, *L. turkestanicus* Knorring	https://wcsp.science.kew.org/
Turkmenistan		*L. balchanicus* Czerniak., *L. gypsaceus* Vved., *L. inebrians* Bunge, *L. cabulicus* Benth.	https://wcsp.science.kew.org/
Uzbekistan	Nuratau and Kyzylkum deserts, Tian-Shan and Pamir-Alay Mountains	*L. acutilobus* (Ledeb.) Fisch. et C. A. Mey., *L. botschantzevii* Kamelin et Zukerv., *L. diacanthophyllus* (Pall.) Benth., *L. gypsaceus* Vved., *L. hirsutissimus* Vved., *L. inebrians* Bunge, *L. knorringianus* Pavlov, *L. kschtutensis* Knorr., *L. nevskii* Knorr., *L. occultiflorus* Rupr., *L. olgae* R. Kamelin, *L. paulsenii* Briq., *L. pubescens* Vved., *L. platyacanthus* Rupr., *L. setulosus* Vved., *L. platycalyx* Schrenk, *L. seravschanicus* Knorr., *L. vvedenskyi* R. Kam. et Zucker.	[13,15]

**Table 2:** Ethnomedicinal uses of different *Lagochilus* species and the used parts.

Species	Country	Parts Used	Traditional Uses	Reference
*L. cabulicus*	Iran	aerial parts	for animals with lung trouble	[23,24]
*L. gypsaceus*	Uzbekistan	aerial parts	hemostatic, sedative effect, decrease in blood pressure, hemorrhage (traumatic, uterine, hemorrhoidal, pulmonary, lung, and nasal), hemophilia	[9,25]
*L. hirtus*	Xinjiang, China	whole plant	styptic, antihemorrhagic, coronary heart diseases, angina pectoris, ulcer, insomnia, amnesia	[10,26]
*L. ilicifolius*	Ningxia, China	whole plant	hemostatic, inflammation, ulcer, hemostasis, spasm, anti-edema, coronary heart disease, angina pectoris, insomnia, dementia	[5,6,7]
*L. inebrians*	Uzbekistan	leaves, stems, fruits, inflorescences	antihemorrhagic, allergic dermatosis, skin illnesses, stomach pain, tranquillizer, intoxicating effect, sedative	[5,8,9]
*L. lanatonodus*	Xinjiang, China	aerial parts	antihemorrhagic, against allergic dermatosis	[6,7,27]
*L. leiacanthus*	Xinjiang, China	whole plant	hemostatic, inflammation, ulcer	[28]
*L. platycalyx*	Uzbekistan	leaves	sedative and hypotensive action	[29]
*L. platyacanthus*	Xinjiang, China	whole plant	antihemorrhagic, coronary heart diseases, angina pectoris, ulcer, insomnia, and amnesia	[10,26]

**Table 3:** Chemical profiling of the isolated compounds from each *Lagochilus* species.

Species	Compounds	References
*L. aucheri*	germacrene D (**107**), α-pinene (**108**), β-bourbonene (**109**)	[18]
*L. cabulicus*	tricetin 3’-methylether (**39**), quercetin (**40**), quercetin 3-O-α-L-rhamnopyranosyl (1→6) β-D-glucopyranoside (**41**), quercetin 3-O-β-D-glucopyranoside (**42**), α-pinene (**108**), *β*-springene (**110**), geranyllinalool (**111**), sitosteryl acetate (**122**), stigmasteryl acetate (**124**), lupeol (**126**)	[24,36]
*L. gypsaceus*	lagochilin (**1**), lagochirsine (**16**), 7- cinnamoyllamalbide (**106**), 5-hydroxy-7,4′-dimethoxyflavone (**48**), daucosterol (**125**), β-sitosterol (**121**), 8-acetylharpagide (**99**)	[37,38,39,40]
*L. hirsutissimus*	lagochilin (**1**), lagohirzidin (**19**), di-*O*-acetyllagohirsin (**18**) [33], lagochirzin, mono- and diacetyllagochirsins (**16–18**) [37,39], stachydrine (**127**), tannins (2.0–3.3%), coumarins (0.3–2.5%), lipids (4.25–8.30%) [41], diterpenoid lactone	[41,42,43]
*L. ilicifolius*	quercetin (**40**), rutin (**43**), myricetin (**44**), isoquercitrin (**45**), kaempferol-3-O-rutinoside (**46**), kaempferol-3-O-β-D-(6″-O-p-coumaryl) glycoside (**47**), (+)-syringaresinol (**138**), scopoletin (**150**), 8-O-acetylharpagide (**99**), harpagide (**100**), ajugoside (**101**), ajujol (**102**), geniposidic acid (**103**), mussaenosidic acid (**104**) and 8-deoxyshanzhiside (**105**), phytol (**142**), 12-hentriacontanol (**143**), octacosanol (**144**), citrusin C (**94**), 4-(1E)-hydroxy-1-prophenyl)-2-methoxyphenol (**95**), 4-acetoxycinnamic acid (**96**), 3-methyl-1,2,3,4-tetrahydroquinoline (**128**), 4-hydroxyisoquinoline (**129**), songoramine (**130**), songorine (**131**), erythro-1-[(4-O-β-D-glucopyranosyl-3-methoxyl)- phenyl]-2-[(5’-methoxyl)-pinoresinol]-propane-1,3-diol (**132**), tortoside C (**133**), sisymbrifolin (**134**)	[5,6,7,21]
*L. inebrians*	lagochilin (**1**), lagochilin and its mono-, di-, tetraacetates (**1–15**), vulgarol and its acetate (**37–38**), 5-hydroxy-4’,7-dimethoxyflavone (**48**), β-sitosterol (**121**), nonacosane (**139**), hentriacontane (**140**), tritriacontane (**141**), 8-O-acetylharpagide (**99**), harpagide (**100**), stachydrine (**127**)	[32,37,40,44,45,46,47,48,49,50,51,52,53,54,55]
*L. kotschyanus*	α-pinene (**108**), myrcene (**115**), *β*-caryophyllene (**116**)	[19]
*L. lanatonodus*	acetovanillone (**72**), androsin (**97**), neolloydosin (98), erythrodiol (**118**), β-sitosterol (**121**), dacosyl ester (**145**), scopoletin (**150**)	[27]
*L. leiacanthus*	15-demethoxyscupolin I (**35**), scupolin I (**36**), 5,2’,6’-trihydroxy-7,8-dimethoxyflavanone (**49**), 5,2’,6’-trihydroxy-6,7,8-trimethoxyflavanone (**50**), 5,2’,6’-trihydroxy-7,8-dimethoxyflavanone-2’-O-β-D-glucoside (**51**), 5,2’-dihydroxy-7,8,6’-trimethoxyflavanone (**53**), 5,2’-dihydroxy-6,7,8,6’-tetramethoxyflavanone (**54**), pinocembrin (**55**), oroxylin A (**56**), chrysin (**57**), 5,6-dihydroxy-7,8-dimethoxyflavone (**58**), isoscutellarein-8-methyl ester (**59**), apigenin (**60**), hispidulin (**61**), 5,2’-dihydroxy- 6,7,8-trimethoxyflavone (**62**), skullcapflavone I (**63**), 5,8- dihydroxy-7,2’-dimethoxyflavone (**64**), 5,2’,6’-trihydroxy- 6,7,8-trimethoxyflavone (**65**), 5,7,2’-trihydroxy-8,6’-dimethoxyflavone (**66**), 5,6,2’-trihydroxy-7,8,6’-trimethoxyflavone (**67**), neobaicalein (**68**), rivularin (**69**), oleanolic acid (**118**), ursolic acid (**119**), vanillin (**70**), *p*-hydroxyacetophenone (**71**), acetovanillone (**72**), dihydroxyskullcapflavanone I (**73**), wogonin (**74**), liquiritin (**75**), viscidulin II 2’-O-glucoside (**76**), 5,2’,6’-trihydroxy-6,7,8- trimethoxyflavone 2’-O-glucoside (**77**)	[28]
*L. macranthus*	caryophyllene oxide (**112**), humulene epoxide II (**113**), viridiflorol (**114**)	[56]
*L. occultiflorus*	laballenic (**146**), octadeca -5,8-dienoic (**147**), eicos -11-enoic (**148**) and eicosa-9,11-dienoic acids (**149**)	[57]
*L. olgae*	lagochirsine (**16**)	[37]
*L. platyacanthus*	lagoditerpenes A-E (**20–24**), (13E)-labd-l3-ene-8α,15-diol (**25**), leojaponins B (**26**), leoheteronin D (**27**), enantioagathic acid (**28**), isocupressic acid (**29**), 7β,13 S-dihydroxylabda-8 (**17**),14-dien-19-oic acid (**30**), 8α,13(R),14(S/R),15-tetrahydroxylabdane (**31**), 15-nor-14-oxolabda-8(17),12E-diene-18-oic acid (**32**), 12β,19-dihydroxymanoyl oxide (**33**), ent-12α,19-dihydroxy-13-epi-manoyl oxide (**34**) (Zhang et al. 2015), rutin (**43**), apigenin (**60**), apigenin-7,4’-dimethylether (**78**), acacetin (**79**), luteolin-7,3’,4’-trimethyl ether (**80**), luteolin-7,4’-dimethyl ether (**81**), diosmetin (**82**), chrysoeriol (**83**), quercetin-3-O-rutinoside-7-O-glucoside (**84**), horridin (**85**), apigenin-6,8-di-C-β-D-glucopyranoside (**86**), isorhamnetin-3-O-rutinoside (**87**), isorhamnetin-3-O-robinobioside (**88**), isorhamnetin-3-O-β-D-glucoside (**89**), isorhamnetin-3-O-rutinoside-4’-O-glucoside (**90**), lavandulifolioside (**91**), 8-O-acetylharpagide (**99**), geniposidic acid (**103**), 1-(4-hydroxy-3-methoxy)-phenyl-2-[4-(1,2,3-trihydroxypropyl)-2-methoxy]-phenoxy- 1,3-propandiol (**135**), (+)-isolarisiresinol 3-α-O-β-D-glucopyranoside (**136**), (-)-isolarisiresinol 3-α-O-β-D-glucopyranoside (**137**)	[58]
*L. platycalyx*	quercetin (**40**), rutin (**43**), myricetin (**44**), kaemferol (**92**), 8-O-acetylharpagide (**99**), harpagide (**100**), stachydrine (**127**)	[29,42,52,53,59]
*L. proskorjacovii*	lagochilin (**1**), tetraacetyllagochilin (**10**), 5-hydroxy-4’,7-dimethoxyflavone (**48**), 5,7-dihydroxy-3,4’-dimethoxyflavone (**93**), β-sitosterol (**121**), nonacosane (**139**)	[12,60,61]
*L. pubescens*	15-mono-O-acetyllagochilin (**3**), 16-mono-O-acetyllagochilin (**4**), 3,18-di-O-acetyllagochilin (**5**), 15,16-di-O-acetyllagochilin (**6**), 3,15,18-tri-O-acetyllagochilin (**7**), 3,16,18-tri-O-acetyllagochilin (**8**), 15,16,18-tri-O-acetyllagochilin (**9**), tetraacetyllagochilin (**10**), 3,18-O-isopropylidenelagochilin-15-acetate (**12**), 3,18-O-isopropylidenelagochilin-15,16-diacetate (**14**), lagochilin (**1**), di-O-isopropylidenelagochilins (**5–6**), 3,18-O-isopropylidenelagochilin (**11**), 16-O-acetyl-3,18-O-isopropylidenelagochilin (**13**), di-O-acetyl-3,18-O-isopropylidenelagochilin (**15**), lagochilin (**1**), 3,18-O-isopropylidenelagochilin (**11**), 5-hydroxy-4’,7-dimethoxyflavone (**48**), β-sitosterol (**121**), nonacosane (**139**), stachydrine (**127**)	[30,31,53,60,62]
*L. setulosus*	lagochilin (**1**), 8-O-acetylharpagide (**99**), harpagide (**100**), lagochirsine (**16**), stachydrine (**127**), ascorbic acid (**151**), β-carotene (**152**)	[37,39,52,53,54,63]
*L. usunachmaticus*	carbohydrates, polysaccharides, pectin, hemicellulose	[64,65]
*L. zeravschanicus*	polysaccharides, pectins, hemicelluloses	[35,64]

**Table 4:** The isolated diterpenes of *Lagochilus* species as described in the literature.

Identified Compounds	Sources	Reference
Lagochilin (**1**)	*L. gypsaceus*, *L. inebrians*, *L. setulosus*, *L. pubescens*, *L. proskorjacovii*, *L. hirsutissimus*	[12,30,32,37,38,41,44,45,46,47,48,49,60,61,63]
3-Mono-O-acetyllagochilin (**2**)	*L. inebrians*	[12,30,32,37,38,41,44,45,46,47,48,49,60,61,62,63]
15-Mono-O-acetyllagochilin (**3**)	*L. inebrians, L. pubescens*	[32,44,45,46,47,48,49,60]
16-Mono-O-acetyllagochilin (**4**)	*L. inebrians, L. pubescens*	[32,44,45,46,47,48,49,60]
3,18-di-O-Acetyllagochilin (**5**)	*L. inebrians, L. pubescens*	[31,32,44,45,46,47,48,49,60,62]
15,16-di-O-Acetyllagochilin (**6**)	*L. inebrians, L. pubescens*	[31,32,44,45,46,47,48,49,60,62]
3,15,18-tri-O-Acetyllagochilin (**7**)	*L. inebrians, L. pubescens*	[32,44,45,46,47,48,49,60]
3,16,18-tri-O-Acetyllagochilin (**8**)	*L. inebrians, L. pubescens*	[32,44,45,46,47,48,49,60]
15,16,18-tri-O-Acetyllagochilin (**9**)	*L. inebrians, L. pubescens*	[32,44,45,46,47,48,49,60]
tetra-Acetyllagochilin (**10**)	*L. inebrians, L. pubescens*	[12,32,44,45,46,47,48,49,60,61]
3,18-O-Isopropylidene-lagochilin (**11**)	*L. inebrians*, *L. pubescens*, *L. proskorjacovii*	[30,31,32,44,45,46,47,48,49,60,62]
3,18-O-Isopropylidene-lagochilin-15-acetate (**12**)	*L. inebrians, L. pubescens*	[30,31,32,44,45,46,47,48,49,60,62]
16-O-Acetyl-3,18-O-isopropylidene-lagochilin (**13**)	*L. inebrians, L. pubescens*	[30,31,32,44,45,46,47,48,49,60,62]
3,18-O-Isopropylidene-lagochilin-15,16-diacetate (**14**)	*L. inebrians, L. pubescens*	[30,31,32,44,45,46,47,48,49,60,62]
di-O-Acetyl-3,18-O-isopropylidene-lagochilin (**15**)	*L. inebrians, L. pubescens*	[30,31,32,44,45,46,47,48,49,60,62]
Lagochirsine (**16**)	*L. hirsutissimus*, *L. setulosus*, *L. gypsaceus*, *L. olgae*	[37,39]
O-Acetyl-lagohirsin (**17**)	*L. hirsutissimus*	[37,39]
di-O-Acetyl-lagohirsin (**18**)	*L. hirsutissimus*	[33,37,39]
Lagohirzidin (**19**)	*L. hirsutissimus*	[33]
Lagoditerpenes A (**20**)	*L. platyacanthus*	[26]
Lagoditerpenes B (**21**)	*L. platyacanthus*	[26]
Lagoditerpenes C (**22**)	*L. platyacanthus*	[26]
Lagoditerpenes D (**23**)	*L. platyacanthus*	[26]
Lagoditerpenes E (**24**)	*L. platyacanthus*	[26]
(13E)-Labd-l3-ene-8α,15-diol (**25**)	*L. platyacanthus*	[26]
Leojaponins B (**26**)	*L. platyacanthus*	[26]
Leoheteronin D (**27**)	*L. platyacanthus*	[26]
Enantioagathic acid (**28**)	*L. platyacanthus*	[26]
Isocupressic acid (**29**)	*L. platyacanthus*	[26]
7β,13 S-Dihydroxylabda-8 (17),14-dien-19-oic acid (**30**)	*L. platyacanthus*	[26]
8α,13(R),14(S/R),15-Tetrahydroxylabdane (**31**)	*L. platyacanthus*	[26]
15-Nor-14-oxolabda-8(17),12E-diene-18-oic acid (**32**)	*L. platyacanthus*	[26]
12β,19-Dihydroxymanoyl oxide (**33**)	*L. platyacanthus*	[26]
ent-12α,19-Dihydroxy-13-epi-manoyl oxide (**34**)	*L. platyacanthus*	[26]
15-Demethoxyscupolin I (**35**)	*L. leiacanthus*	[28]
Scupolin I (**36**)	*L. leiacanthus*	[26]
Vulgarol (**37**)	*L. inebrians*	[44,45,47]
Vulgarol acetate (**38**)	*L. inebrians*	[44,45,47]

**Table 5:** The isolated phenylpropanoids of *Lagochilus* species described in the literature.

Identified compounds	Sources	Reference
Tricetin 3’-methylether (**39**)	*L. cabulicus*	[23,36]
Quercetin (**40**)	*L. cabulicus, L. ilicifolius, L. platycalyx*	[7,23,29,36,59]
Quercetin 3-O-α-L-rhamnopyranosyl (1→6)-β-D-glucopyranoside (**41**)	*L. cabulicus*	[23,36]
Quercetin 3-O-β-D-glucopyranoside (**42**)	*L. cabulicus*	[23,36]
Rutin (**43**)	*L. ilicifolius*, *L. platyacanthus*, *L. platycalyx*	[7,29,58,59]
Myricetin (**44**)	*L. ilicifolius, L. platycalyx*	[7,29,59]
Isoquercitrin (**45**)	*L. ilicifolius*	[7]
Kaempferol-3-O-rutinoside (**46**)	*L. ilicifolius*	[7]
Kaempferol-3-O-β-D-(6″-O-p-coumaryl) glycoside (**47**)	*L. ilicifolius*	[7]
5-Hydroxy-4’,7-dimethoxyflavone (**48**)	*L. gypsaceus L. inebrians L. proskorjacovii, L. pubescens*	[12,30,50,51,60,61]
5,2’,6’-Trihydroxy-7,8-dimethoxyflavanone (**49**)	*L. leiacanthus*	[28]
5,2’,6’-Trihydroxy-6,7,8-trimethoxyflavanone (**50**)	*L. leiacanthus*	[28]
5,2’,6’-Trihydroxy-7,8-dimethoxyflavanone-2’-O-β-D-glucoside (**51**)	*L. leiacanthus*	[28]
5,2’,6’-Trihydroxy-6,7,8-trimethoxyflavanone-2’-O-β-D-glucoside (**52**)	*L. leiacanthus*	[28]
5,2’-Dihydroxy-7,8,6’-trimethoxyflavanone (**53**)	*L. leiacanthus*	[28]
5,2’-Dihydroxy-6,7,8,6’-tetramethoxyflavanone (**54**)	*L. leiacanthus*	[28]
Pinocembrin (**55**)	*L. leiacanthus*	[28]
Oroxylin A (**56**)	*L. leiacanthus*	[28]
Chrysin (**57**)	*L. leiacanthus*	[28]
5,6-Dihydroxy-7,8-dimethoxyflavone (**58**)	*L. leiacanthus*	[28]
Isoscutellarein-8-methyl ester (**59**)	*L. leiacanthus*	[28]
Apigenin (**60**)	*L. leiacanthus, L. platyacanthus*	[28,58]
Hispidulin (**61**)	*L. leiacanthus*	[28]
5,2’-Dihydroxy-6,7,8-trimethoxyflavone (**62**)	*L. leiacanthus*	[28]
Skullcapflavone I (**63**)	*L. leiacanthus*	[28]
5,8- Dihydroxy-7,2’-dimethoxyflavone (**64**)	*L. leiacanthus*	[28]
5,2’,6’-Trihydroxy- 6,7,8-trimethoxyflavone (**65**)	*L. leiacanthus*	[28]
5,7,2’-Trihydroxy-8,6’-dimethoxyflavone (**66**)	*L. leiacanthus*	[28]
5,6,2’-Trihydroxy-7,8,6’-trimethoxyflavone (**67**)	*L. leiacanthus*	[28]
Neobaicalein (**68**)	*L. leiacanthus*	[28]
Rivularin (**69**)	*L. leiacanthus*	[28]
Vanillin (**70**)	*L. leiacanthus*	[28]
*p*-Hydroxyacetophenone (**71**)	*L. leiacanthus*	[28]
Acetovanillone (**72**)	*L. leiacanthus, L. lanatonodus*	[27,28]
Dihydroxyskullcapflavanone I (**73**)	*L. leiacanthus*	[28]
Wogonin (**74**)	*L. leiacanthus*	[28]
Liquiritin (**75**)	*L. leiacanthus*	[28]
Viscidulin II 2’-O-glucoside (**76**)	*L. leiacanthus*	[28]
5,2’,6’-Trihydroxy-6,7,8- trimethoxyflavone 2’-O-glucoside (**77**)	*L. leiacanthus*	[28]
Apigenin-7,4’-dimethylether (**78**)	*L. platyacanthus*	[58]
Acacetin (**79**)	*L. platyacanthus*	[58]
Luteolin-7,3’,4’-trimethyl ether (**80**)	*L. platyacanthus*	[58]
Luteolin-7,4’-dimethyl ether (**81**)	*L. platyacanthus*	[58]
Diosmetin (**82**)	*L. platyacanthus*	[58]
Chrysoeriol (**83**)	*L. platyacanthus*	[58]
Quercetin-3-O-rutinoside-7-O-glucoside (**84**)	*L. platyacanthus*	[58]
Horridin (**85**)	*L. platyacanthus*	[58]
Apigenin-6,8-di-C-β-D-glucopyranoside (**86**)	*L. platyacanthus*	[58]
Isorhamnetin-3-O-rutinoside (**87**)	*L. platyacanthus*	[58]
Isorhamnetin-3-O-robinobioside (**88**)	*L. platyacanthus*	[58]
Isorhamnetin-3-O-β-D-glucoside (**89**)	*L. platyacanthus*	[58]
Isorhamnetin-3-O-rutinoside-4’-O-glucoside (**90**)	*L. platyacanthus*	[58]
Lavandulifolioside (**91**)	*L. platyacanthus*	[58]
Kaemferol (**92**)	*L. platycalyx*	[29,59]
5,7-Dihydroxy-3,4’-dimethoxyflavone (**93**)	*L. pubescens*	[12,30,60,61]
Citrusin C (**94**)	*L. ilicifolius*	[5,21]
4-(1E)-Hydroxy-1-prophenyl)-2-methoxyphenol (**95**)	*L. ilicifolius*	[5,21]
4-Acetoxycinnamic acid (**96**)	*L. ilicifolius*	[5,21]
Androsin (**97**)	*L. lanatonodus*	[27]
Neolloydosin (**98**)	*L. lanatonodus*	[27]

**Table 6:** The hemostatic effect of different *Lagochilus* species extracts and their isolated components.

Species	Tested Sample	Test Type	Main Finding	Reference
*Lagochilus* species	*Lagochilus* preparations	*in vivo*	increased the coagulation ability of the blood both by activating plasma and cellular blood coagulation factors and by depressing the anticoagulant system, and also have a suppressive effect on plasma fibrinolytic activity; accelerating the blood coagulation process, reducing vascular permeability, lowering blood pressure, sedative and analgesic effects	[25,74,75,76,77]
*Lagochilus* species	lagochiline acetic acid ester	*in vivo*	showed sedative properties; LD_50_ for mice was 3.6 g/kg. Injected subcutaneously into rabbits in doses of 0.05 g/kg, it hastened the process of blood coagulation by 30–40%; it affected a reduction in the bleeding time and the volume of blood lost	[78]
*Lagochilus* species	*Lagochilus* infusion and pure lagochilin (**1**)	*in vivo*	in doses of 0.05 g/kg in dogs shortened the blood-clotting and prothrombin times by 40–60%; the effects were even more pronounced in dicoumarol hemophilia.	[79]
*L. inebrians*	Lagoden drug (5% solution of lagohirisine sodium salt) has been developed and approved for public use	*in vivo*	Lagoden is prescribed for the treatment and prevention of acute and chronic bleeding (gastrointestinal, hemorrhoidal, pulmonary, uterine, etc.), parenchymal hemorrhage (renal, splenic, brain), capillary, and other bleeding, for surgical interventions in otolaryngology practice (for tonsillary tumors, juvenile nasopharyngeal angiofibroma), microsurgery on the ear, etc., in dentistry for removing teeth, cysts, granulomas, during surgery of the gastrointestinal tract, prostate adenomectomy, gynecological operations on the uterus, including bleeding associated with a violation of the blood coagulation system	Pharmacological Committee of the Republic of Uzbekistan (registration Certificate N 01/195/1 of 08.05.2001)
*L. inebrians*	Inebrin drug (extractive substances)	*in vivo*	recommended in the form of tablets for the treatment of chronic uterine, nasal, gastrointestinal, and other bleeding	[80,81,82]
*L. inebrians*	compound **1** and **16** and their natural and synthetic derivatives (with cellulose acetate, mono-, di-, tri- and tetrasodium succinates, supramolecular complexes of lagochilin with glycyrrhizic acid and its monoammonium salt, etc.)	*in vivo*	hemostatic activity	[11,39,71,82,83]
*L. usunachmaticus*	polysaccharides and carbohydrates isolated from the epigeal part of the plant	*in vivo*	possessed a marked direct-action activity and exceeded heparin during affection	[35,64,65]
*L. setulosus*	Setulin (obtained from a dry extract of the plant)	*in vivo*	Setulin hemostatic drug excipient in rabbits in a dose of 50 mg/kg causes the expressed hemostatic effect associated with the activation of thromboplastin formation and transformation of prothrombin into thrombin owing to the acceleration of contact and phospholipid coagulation starting mechanisms (I and II phases of blood coagulation). At 60–90 min after introduction, Setulin completely removes the hypocoagulative effect of heparin	[84]
*L. diacanthophyllus*	H_2_O and 95% EtOH extracts	*in vivo*	EtOH extract significantly shorten the clotting time of mice, bleeding time, show some hemostatic activity	[85]
*L. platyacanthus*	lagoditerpenes **20**, **21** and **24**	*in vivo*	lagoditerpenes **20**, **21**, and **24** showed that moderate hemostatic activities by shortening the values of activated partial thromboplastin time (APTT). These compounds were able to shorten the values of APTT, while the values of prothrombin time and thromboplastin time were not obviously shorted	[58]
*L. lanatonodus, L. diacanthophyllus, L. platyacanthus L. hirtus, L. ilicifolius*	H_2_O and EtOH extracts	*in vivo*	*L. lanatonodus* and *L. diacanthophyllus* showed better hemostatic activities among five species. The extracts of *L. lanatonodus* and *L. diacanthophyllus* showed dose-dependent hemostatic effects. Both H_2_O and EtOH extracts of *L. lanatonodus* at 400 mg/kg in rats greatly reduced the blood clotting time and tail bleeding time	[10]

**Table 7:** The anti-inflammatory activities of different *Lagochilus* species extracts.

Species	Tested Sample	Test Performed	Main Finding	Reference
*L. inebrians*	5% infusion of leaves	*in vivo*	from 10 guinea pigs, 8 were less sensitive to *L. inebrians*	[86]
*L. inebrians*	tincture of aerial parts	*in vivo*	most active inhibitor of edema in frog legs among the tested samples	[87]
*L. diacanthophyllus*	H_2_O extract or 95% EtOH extract	*in vivo*	extracts can suppress xylene-induced ear edema in mice, showed some anti-inflammatory activity in vivo	[85]
*L. diacanthophyllus*	H_2_O extract or 95% EtOH extract	*in vitro*	EtOH extract significantly inhibit macrophage release of NO, TNF-α, IL-6, showed strong in vitro anti-inflammatory activity	[85]
five *Lagochilus* species (*L. hirtus*, *L. platyacanthus*, *L. lanatonodus*, *L. diacanthophyllus*, and *L. ilicifolius*)	H_2_O and EtOH extracts	*in vivo*	the extracts of *L. lanatonodus* and *L. diacanthophyllus* showed strong inhibitory effects on the acute phase of inflammation in both xylene-induced ear edema mouse model and carrageenan-induced paw edema rat model. Aqueous extract of *L. lanatonodus* showed the best anti-inflammatory activities among the five *Lagochilus* species. *L. lanatonodus* extracts can significantly modulate inflammatory indexes, that is, lower NO, MDA, and PEG2 and elevate SOD. *L. platyacanthus, L. hirtus,* and *L. ilicifolius,* exhibited little potency in alleviating edema	[10]

**Table 8:** The antibacterial activities of the components from *Lagochilus* species.

Species	Tested sample	Microorganism	Main finding	Reference
*L. ilicifolius*	EtOH, petroleum ether, CHCl_3_, EtOAc, *n*-BuOH extracts, water remainder, and total alkaloids	*S. aureus*, *B. subtilis*, *B. cereus*, *E. coli*	EtOAc ext. inhibited *B. subtilis* (3.0 ± 0.1 mm), EtOH, EtOAc, *n*-BuOH extracts inhibited *B. cereus* (5.0 ± 0.1 mm) (at 100 µg/disc concentration)	[5,6,7,21]
*L. kotschyanus*	essential oils of flowers and leaves	*S. aureus*, *S. pyogenes*, *S. agalactia,B. anthracis*, *K. pneumoniae*, *P. aeruginosa*	flowers oil showed strong inhibitory activity against *S. pyogenes*, *S. agalactia*, *B. anthracis*, *K. pneumoniae*, and *P. aeruginosa;* leaves oil only showed inhibitory activity against *S. pyogenes*	[19]
*L. cabulicus*	H_2_O and EtOH extracts	*S. aureus* ATCC 6538; *E. coli* ATCC 11229; *B. subtilis* ATCC 6633; *P. aeriginosa* ATCC 9027	only EtOH extract inhibited *S. aureus* ATCC 6538 at 0.5 mg/mL	[24]
*L. acutilobus*, *L. gypsaceus*, *L. inebrians*, *L. olgae*, *L. proskorjakovii*, *L. setulosus*, *L. vvedenskyi*	compounds lagochilin (**1**), 5-hydroxy-4’,7-dimethoxyflavone (**48**), 8-O-acetylharpagide (**99**), β-sitosterol (**121**), stigmasterol (**123**), daucosterol (**125**), MeOH extracts obtained from the aerial parts of plants	*S. aureus* ATCC 25923, *B. subtilis* RKMUz 5, *P. aeruginosa* ATCC 27879, *E. coli* RKMUz 221, *C. albicans* RKMUz 247	compounds **1**, **48**, **99**, **121**, **123**, **125** were inactive against the tested microorganisms; *B. subtilis* (9.12 ± 0.13 mm for MeOH ext. of *L. proskorjakovii* and 9.04 ± 0.10 mm for MeOH ext. of *L. olgae*) MeOH extracts of *L. inebrians, L. olgae,* and *L. proskorjakovii* were more active against *B. subtilis* with MIC = 125 μg/mL	[88]

**Table 9:** Enzyme inhibitory activities of the components from *Lagochilus* species.

Species	Tested Sample	Enzyme	Main Finding	Reference
*L. cabulicus*	EtOAc and MeOH ext	urease	the extracts showed no Jack bean urease inhibitory activity	[34]
*L. inebrians, L. setulosus, L. gypsaceus*	essential oils	AChE, BChE, tyrosinase, glucosidase, amylase	no inhibition was observed by *L. gypsaceus* EO; *L. setulosus* EO had no effect against glucosidase; tyrosinase inhibitory activity of EO followed *L. inebrans*>*L. setulosus*>*L. gypsaceus; L. setulosus* EO and *L. inebrans* EO showed amylase and glucosidase inhibitory effects	[69]
*L. acutilobus*, *L. gypsaceus*, *L. inebrians*, *L. olgae*, *L. proskorjakovii*, *L. setulosus*, *L. vvedenskyi*	MeOH extracts and compounds lagochilin (**1**), 5-hydroxy-4’,7-dimethoxyflavone (**48**), 8-O-acetylharpagide (**99**), β-sitosterol (**121**), daucosterol (**125**)	AChE, BChE, tyrosinase, glucosidase, amylase	compound **48** exhibited the strongest inhibitory effects on both AChE and BChE; the highest tyrosinase inhibitory effect was found for MeOH ext of *L. inebrians* (from Djizzakh) and **48**; MeOH ext of *L. acutilobus* and **48** showed the best amylase inhibitory effects; MeOH ext of *L. inebrians* and **48** exhibited stronger glucosidase inhibitory effects; compound **99** had the weakest effect on tested enzymes	[40,88]

## Supplementary Materials

The following are available online at https://www.mdpi.com/2223-7747/10/1/132/s1, The chemical structures isolated from *Lagochilus* species are available online, alongside Figures S1–S11. **Figure S1**. Chemical structures of isolated diterpenes from the genus *Lagochilus*, Figure S2. Chemical structures of isolated phenylpraponoids from the genus *Lagochilus,* Figure S3. Chemical structures of isolated iridoids from the genus *Lagochilus,* Figure S4. Chemical structures of some isolated terpenoids from the genus *Lagochilus,* Figure S5. Chemical structures of isolated triterpenoids from the genus *Lagochilus,* Figure S6. Chemical structures of isolated steroids from the genus *Lagochilus,* Figure S7. Chemical structures of isolated alkaloids from the genus *Lagochilus,* Figure S8. Chemical structures of isolated lignans from the genus *Lagochilus,* Figure S9. Chemical structures of isolated aliphatic alkanes and alcohols from the genus *Lagochilus,* Figure S10. Chemical structures of some of isolated lipids from the genus *Lagochilus,* Figure S11. Chemical structures of other isolated compounds from the genus *Lagochilus.*
