# Supplementary material for: The Genus Lagochilus (Lamiaceae): A Review of Its Diversity, Ethnobotany, Phytochemistry, and Pharmacology
Source: Plants (Basel). 2021 Jan 11;10(1):132. doi: 10.3390/plants10010132 (PMC7826601; doi:10.3390/plants10010132)
Supplement: Supplementary file 1 [file plants-10-00132-s001.pdf]

Supporting Information

The Genus *Lagochilus* (Lamiaceae): A Review of Its Diversity, Ethnobotany, Phytochemistry and Pharmacology

Nilufar Z. Mamadalieva [1,2,*], Davlat Kh. Akramov [1], Ludger A. Wessjohann [2], Hidayat Hussain [2], Chunlin Long [3,4], Komiljon Sh. Tojibaev [5], Elham Alshammari [6], Mohamed L. Ashour [7,8] and Michael Wink [9,*]

[1] Institute of the Chemistry of Plant Substances of the Academy Sciences of Uzbekistan, Mirzo Ulugbek Str 77, Tashkent 100170, Uzbekistan; a.davlat@inbox.ru
[2] Department of Bioorganic Chemistry, Leibniz Institute of Plant Biochemistry, Weinberg 3, 06120 Halle (Saale), Germany; ludger.wessjohann@ipb-halle.de (L.A.W); hidayat.hussain@ipb-halle.de (H.H.)
[3] College of Life and Environmental Sciences, Minzu University of China, Beijing 100081, China
[4] Key Laboratory of Ethnomedicine (Minzu University of China), Ministry of Education, Beijing 100081, China; long@mail.kib.ac.cn
[5] Institute of Botany of the Academy Sciences of Uzbekistan, Durmon Yuli Str 32, Tashkent 100125, Uzbekistan; ktojibaev@mail.ru
[6] Department of Pharmacy Practice, College of Pharmacy, Princess Nourah bint Abdulrahman University, Riyadh 11671, Saudi Arabia; ejalshammari@pnu.edu.sa
[7] Department of Pharmaceutical Sciences, Pharmacy Program, Batterjee Medical College, Jeddah 21442, Saudi Arabia
[8] Department of Pharmacognosy, Faculty of Pharmacy, Ain Shams University, Cairo 11566, Egypt; ashour@pharma.asu.edu.eg
[9] Department of Pharmaceutical Biology, Institute of Pharmacy and Molecular Biotechnology, Heidelberg University, 69120 Heidelberg, Germany

* Correspondence: wink@uni-heidelberg.de (M.W.); nmamadalieva@yahoo.com (N.Z.M); Tel.: +49-6221-54-4880 (M.W.); Fax: +49-6221-54-4884 (M.W.)

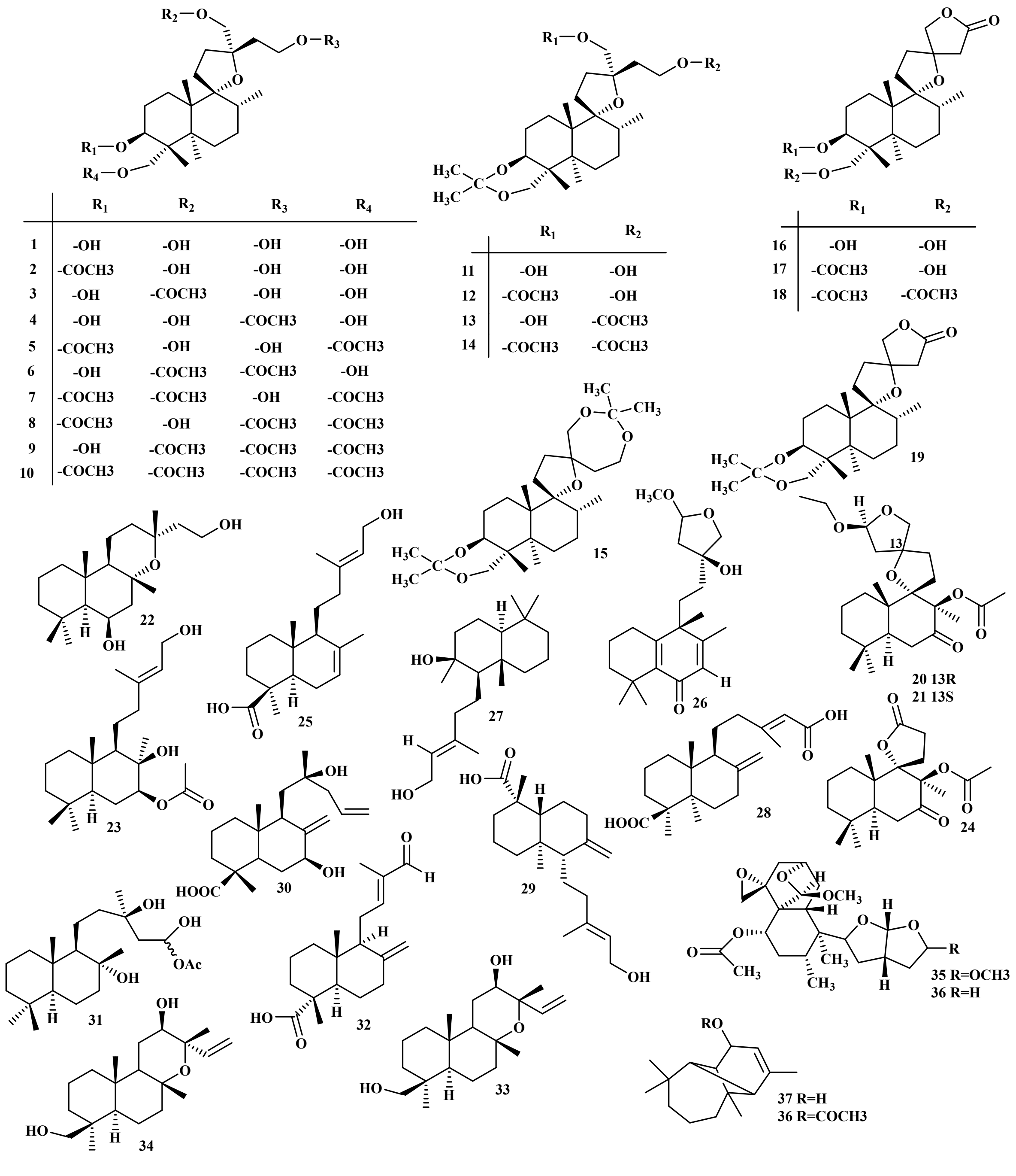

	R1	R2	R3	R4
1	-OH	-OH	-OH	-OH
2	-COCH3	-OH	-OH	-OH
3	-OH	-COCH3	-OH	-OH
4	-OH	-OH	-COCH3	-OH
5	-COCH3	-OH	-OH	-COCH3
6	-OH	-COCH3	-COCH3	-OH
7	-COCH3	-COCH3	-OH	-COCH3
8	-COCH3	-OH	-COCH3	-COCH3
9	-OH	-COCH3	-COCH3	-COCH3
10	-COCH3	-COCH3	-COCH3	-COCH3

	R1	R2
11	-OH	-OH
12	-COCH3	-OH
13	-OH	-COCH3
14	-COCH3	-COCH3

	R1	R2
16	-OH	-OH
17	-COCH3	-OH
18	-COCH3	-COCH3

Figure S1. Chemical structures of isolated diterpenes from the genus *Lagochilus*

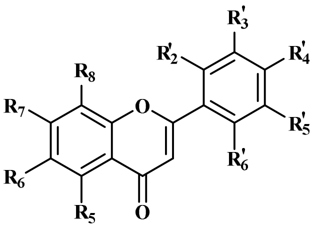

	R_5	R_6	R_7	R_8	R'_2	R'_3	R'_4	R'_5	R'_6
39	-OH	-H	-OH	-H	-H	-OH	-OH	-OCH3	-H
40	-OH	-H	-OH	-H	-H	-OH	-OH	-H	-H
44	-OH	-H	-OH	-H	-H	-OH	-OH	-OH	-H
48	-OH	-H	-OCH3	-H	-H	-H	-OCH3	-H	-H
49	-OH	-H	-OCH3	-OCH3	-OH	-H	-H	-H	-OH
50	-OH	-OCH3	-OCH3	-OCH3	-OH	-H	-H	-H	-OH
53	-OH	-H	-OCH3	-OCH3	-OH	-H	-H	-H	-OH
54	-OH	-OCH3	-OCH3	-OCH3	-OH	-H	-H	-H	-OH
56	-OH	-OCH3	-OH	-H	-H	-H	-H	-H	-H
57	-OH	-H	-OH	-H	-H	-H	-H	-H	-H
58	-OH	-OH	-OCH3	-OCH3	-H	-H	-H	-H	-H
59	-OH	-H	-OH	-OCH3	-H	-H	-OH	-H	-H
60	-OH	-H	-OH	-H	-H	-H	-OH	-H	-H
61	-OH	-OCH3	-OH	-H	-H	-H	-OH	-H	-H
62	-OH	-OCH3	-OCH3	-OCH3	-OH	-H	-H	-H	-H
63	-OH	-H	-OCH3	-OCH3	-OH	-H	-H	-H	-H
64	-OH	-H	-OCH3	-OH	-OCH3	-H	-H	-H	-H
65	-OH	-OCH3	-OCH3	-OCH3	-OH	-OH	-OH	-OH	-OH
66	-OH	-H	-OH	-OCH3	-OH	-H	-H	-H	-OCH3
67	-OH	-OH	-OCH3	-OCH3	-OH	-H	-H	-H	-OCH3
68	-OH	-OCH3	-OCH3	-OCH3	-OH	-H	-H	-H	-OCH3
69	-OH	-H	-OCH3	-OCH3	-OH	-H	-H	-H	-OCH3
74	-OH	-H	-OH	-OCH3	-H	-H	-H	-H	-H
78	-OH	-H	-OCH3	-H	-H	-H	-OCH3	-H	-H
79	-OH	-H	-OH	-H	-H	-H	-OCH3	-H	-H
80	-OH	-H	-OCH3	-H	-H	-OCH3	-OCH3	-H	-H
81	-OH	-H	-OCH3	-H	-H	-OH	-OCH3	-H	-H
82	-OH	-H	-OCH3	-H	-H	-OH	-OCH3	-H	-H
83	-OH	-H	-OH	-H	-H	-OCH3	-OH	-H	-H

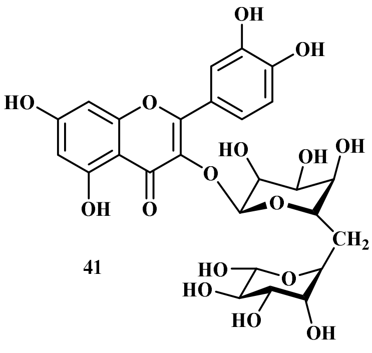

41

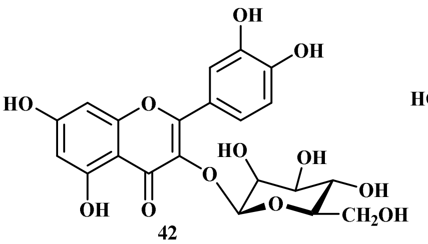

42

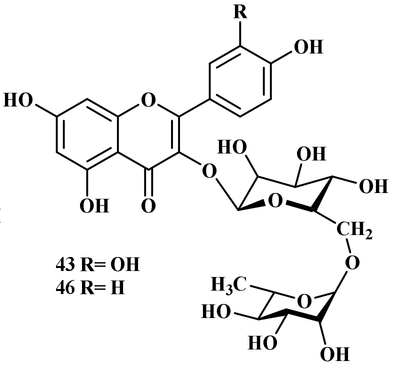

43 R= OH
46 R= H

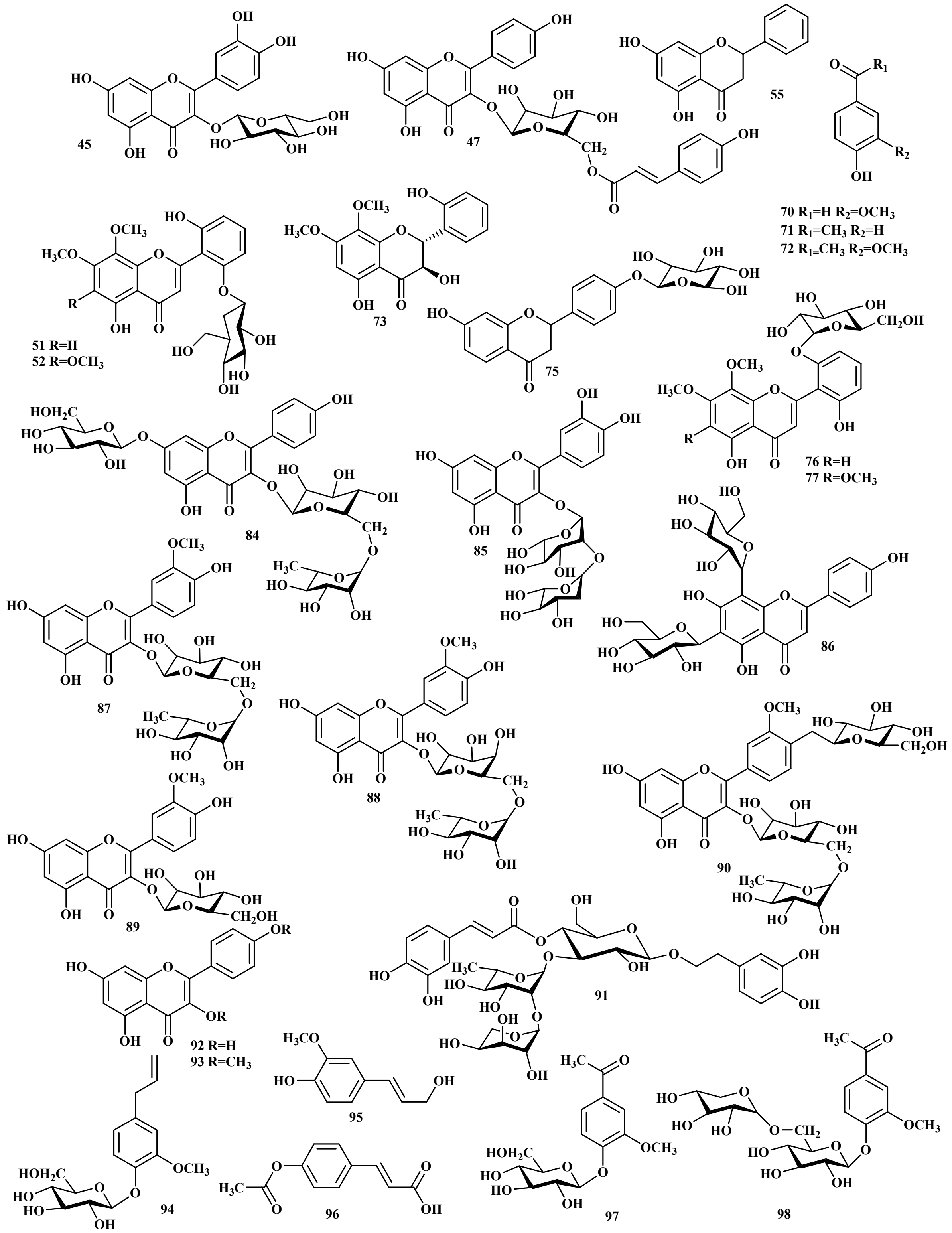

Figure S2. Chemical structures of isolated phenylpraponoids from the genus *Lagochilus*

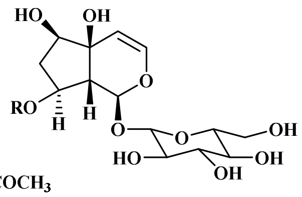

99 R=$COCH_3$
100 R= H

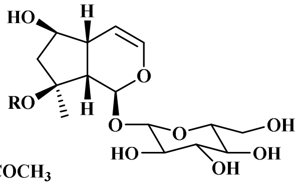

101 R=$COCH_3$
102 R= H

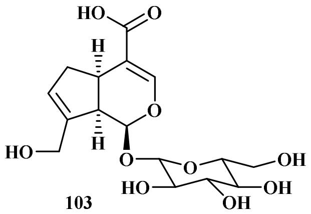

103

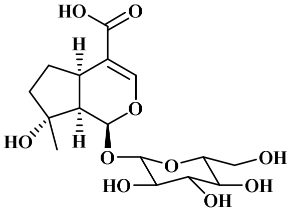

104

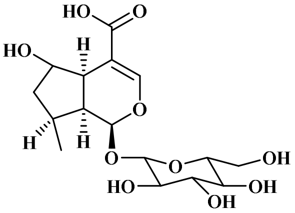

105

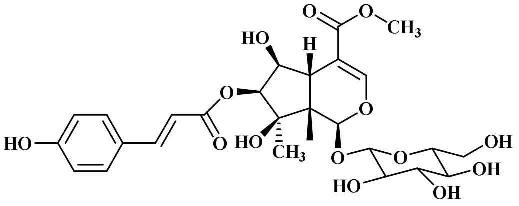

106

Figure S3. Chemical structures of isolated iridoids from the genus *Lagochilus*

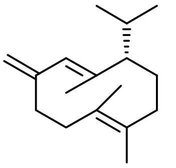

107

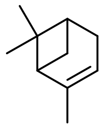

108

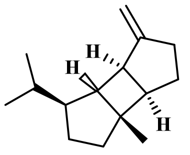

109

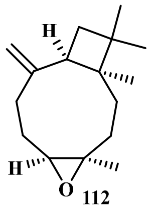

112

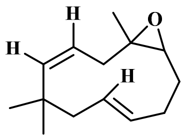

113

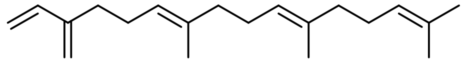

110

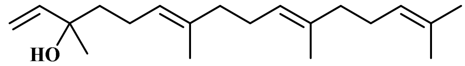

111

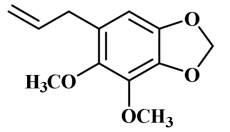

117

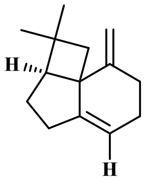

116

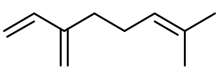

115

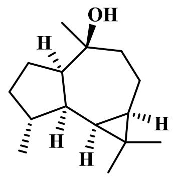

114

Figure S4. Chemical structures of some isolated terpenoids from the genus *Lagochilus*

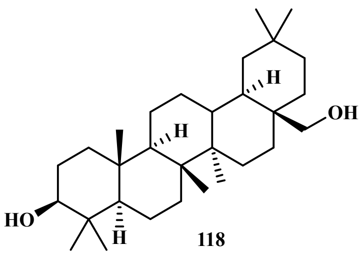

118

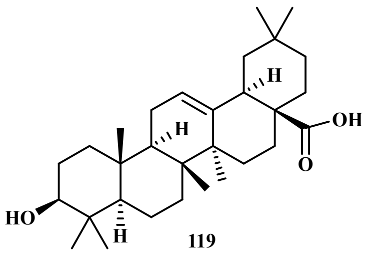

119

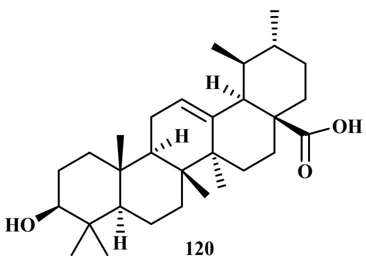

120

Figure S5. Chemical structures of isolated triterpenoids from the genus *Lagochilus*

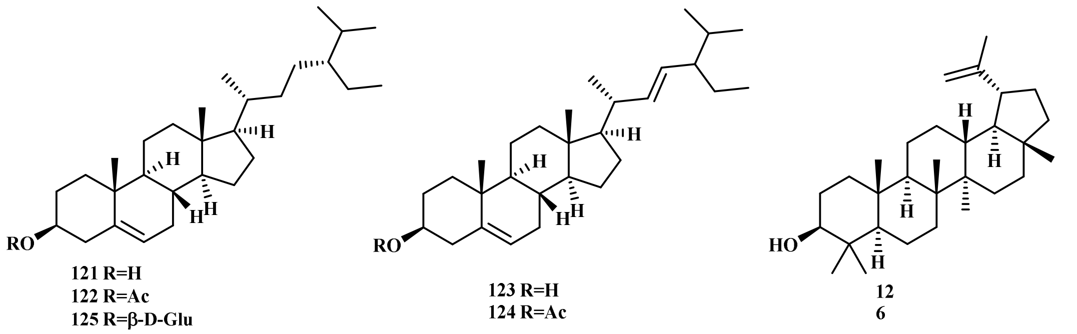

Figure S6. Chemical structures of isolated steroids from the genus *Lagochilus*

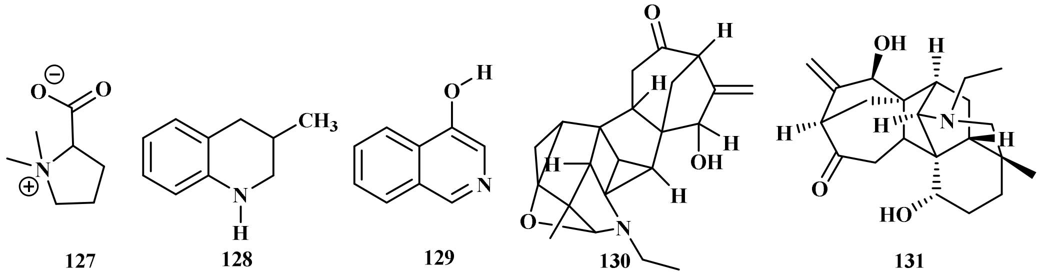

Figure S7. Chemical structures of isolated alkaloids from the genus *Lagochilus*

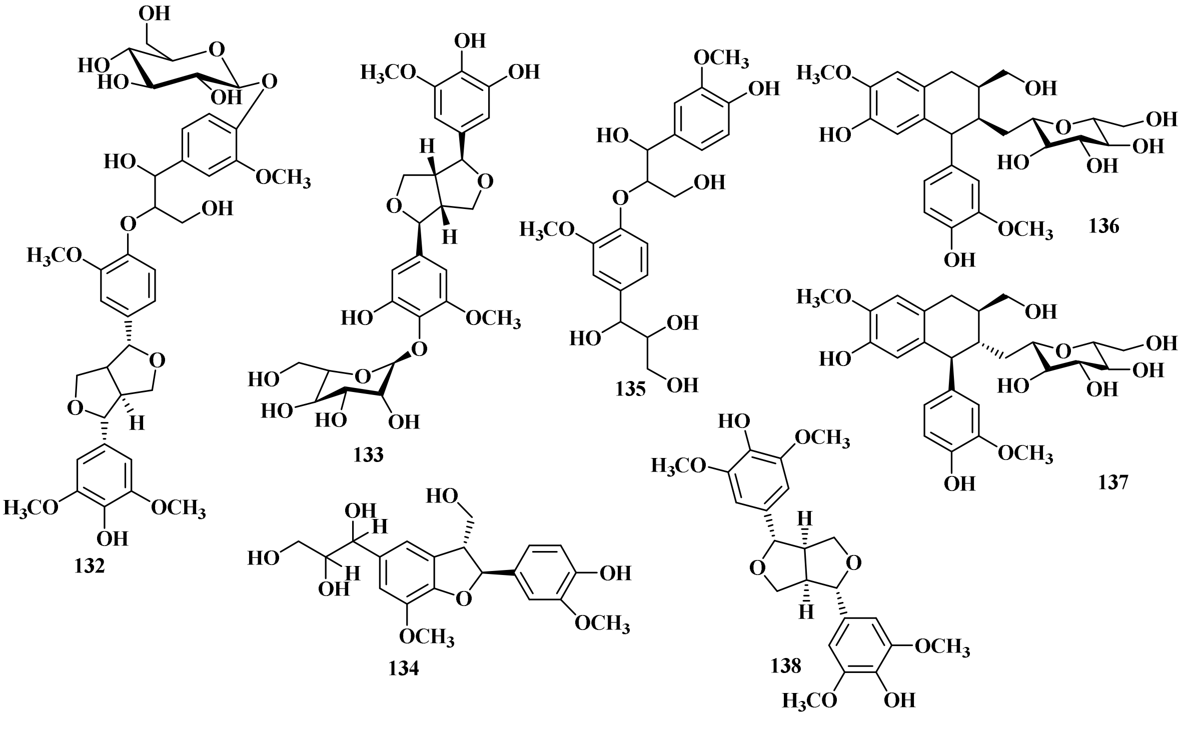

Figure S8. Chemical structures of isolated lignans from the genus *Lagochilus*

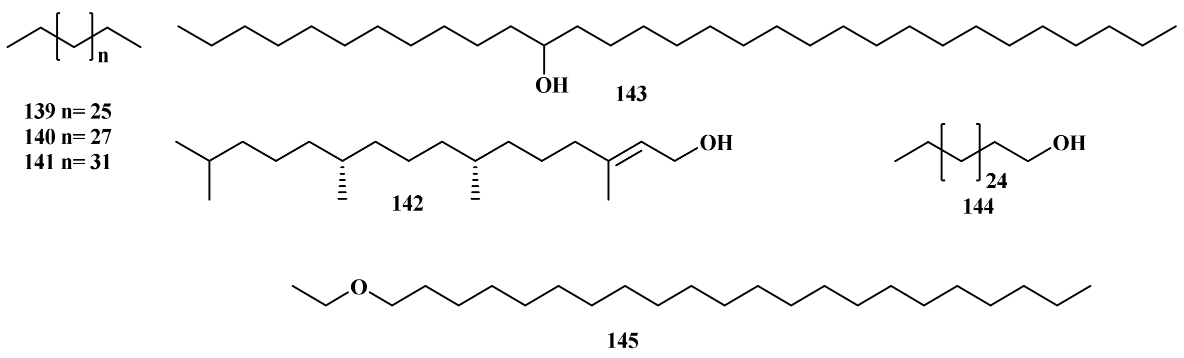

Figure S9. Chemical structures of isolated aliphatic alkanes and alcohols from the genus *Lagochilus*

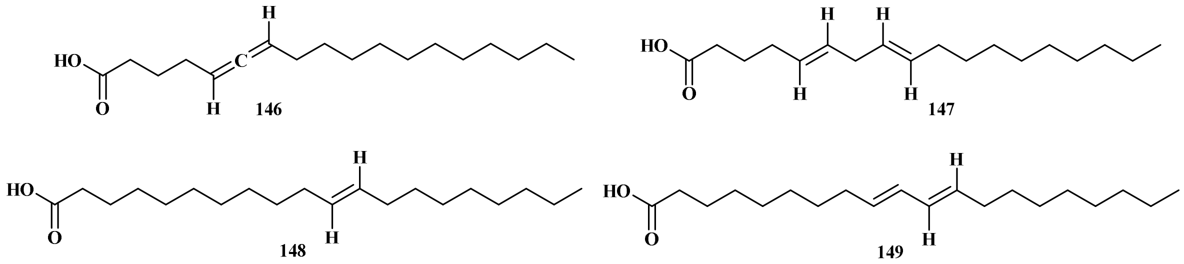

Figure S10. Chemical structures of some of isolated lipids from the genus *Lagochilus*

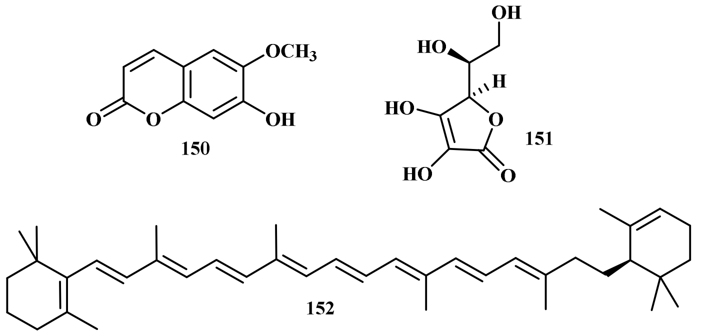

Figure S11. Chemical structures of other isolated compounds from the genus *Lagochilus*
